# Determination of the Generation Efficiency and Reductive Reactivity of the Upconverted Hot Electrons from Mn-Doped Quantum Dots under Weak Visible Light

**DOI:** 10.1021/jacs.5c12004

**Published:** 2025-10-02

**Authors:** Connor Orrison, Ian C. Schulze, Zefan Zhang, Dallas Freitas, Ian Murray, Xin Yan, Christian Hilty, Dong Hee Son

**Affiliations:** 1 Department of Chemistry, 14736 Texas A&M University, College Station, Texas 77843, United States; 2 Department of Physics and Astronomy, Texas A&M University, College Station, Texas 77843, United States; 3 Center for Nanomedicine, Institute for Basic Science and Graduate Program of Nano Biomedical Engineering, Advanced Science Institute, Yonsei University, Seoul 03722, Republic of Korea

## Abstract

Hot electrons generated via Mn-mediated Auger upconversion in Mn-doped quantum dots (QDs) have been shown to be highly effective for reduction reactions that require high reduction potentials and long-range electron transfer. Due to their high energy, these electrons can drive reduction reactions in the solution phase through multiple pathways, including interfacial hot electron transfer on the QD surface and reduction by presolvated and solvated electrons within the solvent. Despite their demonstrated ability to drive challenging reactions, the efficiency of generating upconverted hot electrons under continuous-wave (cw) visible light excitation remains largely unknown. Here, we quantified the quantum yield of hot electron generation from Mn-doped CdSSe/ZnS QDs under continuous white light (455 nm) in aqueous media using the reductive dechlorination of monochloroacetate (MCA) as a hot electron-selective reaction. We found that the quantum yield for hot electron upconversion can reach 35–40% at an excitation intensity of ∼0.1 W/cm^2^, highlighting the high efficiency of Mn-doped QDs as a source of hot electrons. Furthermore, we observed a dependence of the product yield on the photoexcitation history, suggesting a possible excitation-induced surface charge affecting the local concentration of reactants near the QDs, thereby altering the reaction kinetics. These findings provide important insights into the design of more efficient Mn-doped QD structures and reaction conditions for hot electron-enabled photocatalysis using visible light-driven hot electron sources.

## Graphical abstract

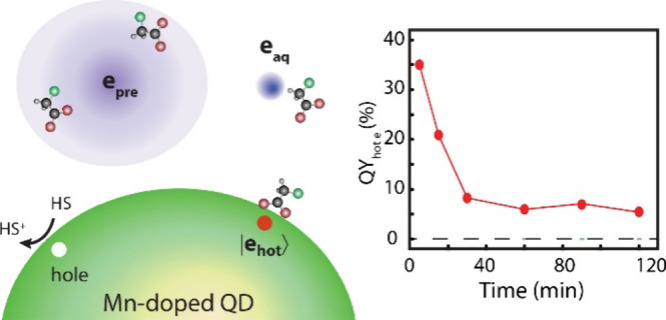

## Introduction

Photogenerated hot electrons carrying excess energy above the conduction band edge in semiconductors and metals have attracted much interest due to their advantages over thermalized band-edge electrons in photovoltaics^1–4^ and photocatalysis.^5–10^ The excess energy of hot electrons facilitates electron transfer across the high-energy barriers over longer distances, resulting in more efficient charge extraction or reduction reactions in these applications. Moreover, sufficiently energetic hot electrons injected into liquid surroundings can generate solvated electrons with a much longer lifetime than hot electrons (e.g., ∼μs in water), which can enable solvated electron-induced reactions in addition to hot electron transfer prior to solvation.^11–14^

Recently, hot electrons generated via the visible light-driven Auger upconversion process in semiconductor quantum dots (QDs) have demonstrated their ability to perform reactions requiring high reduction potentials or to perform long-range hot electron transfer across the barrier.^14–22^ Notably, hot electrons generated from Mn-doped QDs via Mn-mediated Auger upconversion have been shown not only to generate photoelectron emission above the vacuum level from their higher-energy subpopulation in vacuum but also to produce solvated electrons in liquid media.^12,14,23–25^ The average energy of these hot electrons, produced via the Mn-mediated upconversion process, is estimated to be a small fraction of an eV below the vacuum level, equivalent to approximately −4 V vs NHE or more negative depending on the size of the QDs in the case of Mn-doped CdS/ZnS QDs.^23,26^ Therefore, the energy of the upconverted hot electrons is sufficiently high not only to perform hot electron transfer upon generation but also to be injected into liquid surroundings and subsequently form solvated electrons. This opens the highly reactive solvated electron-induced reduction channel that is well known from the earlier studies in radiation chemistry.^27,28^ Furthermore, the long excited state lifetime of Mn^2+^ (e.g., several milliseconds in II–VI semiconductor hosts), acting as an intermediate state for upconversion, enables the generation of hot electrons under relatively low-intensity, continuous-wave (cw) excitation (e.g., 0.1–1 W/cm^2^).^29–31^ These advantages make the upconverted hot electrons generated from Mn-doped QDs attractive for practical hot electron- and solvated electron-based photocatalysis.

In aqueous media, the hot electrons injected into water from the photoexcited QDs were reported to become solvated in <1 ps forming the hydrated electron (e_aq_), similar to the time scale of solvation of UV-photoionized electrons from molecular and ionic precursors.^32^ In earlier studies on e_aq_ produced from UV photoionization of water and anionic precursors, “quasi-free electrons” or “presolvated electrons” were frequently mentioned as the higher-energy precursor states of e_aq_.^32–34^ These species were often more finely distinguished depending on their formation mechanism and specific stage along the path to solvation. Here, we collectively refer to all precursor states of e_aq_ as “presolvated hot electrons (e_pre_)” to distinguish their collective reductive chemistry from that of e_aq_. Several earlier studies have shown that e_pre_ can also perform efficient reduction reactions via static scavenging, although their reactive chemistry has not been investigated as extensively as that of e_aq_.^34^ Despite their short lifetime (e.g., < 1 ps), the larger spatial extent of e_pre_ (e.g., radius >3 nm as opposed to ∼0.3 nm for e_aq_) can enable reduction reactions in addition to the reduction channel by e_aq_.^34,35^ The static scavenging of e_pre_ becomes more effective at high concentrations of the electron acceptors capable of forming encounter complexes with many electron acceptors. When upconverted hot electrons generated from Mn-doped QDs are used for reduction, reactants bound to the QD surface can also undergo reduction via direct interfacial hot electron transfer before the solvation of hot electrons. Therefore, upconverted hot electrons from Mn-doped QDs can perform reduction via three distinct pathways involving direct interfacial hot electron transfer, e_pre_ and e_aq_. This is also a difference from the reduction by e_pre_ and e_aq_ derived from the ionization of molecular and ionic precursors, which lack an interfacial hot electron transfer component.

While several studies have demonstrated the enhanced reduction capability of the upconverted hot electrons generated via cw visible excitation of Mn-doped QDs, the quantum yield of generating hot electrons, a key metric of the QD’s photocatalytic performance, remains largely unknown.^17–20,22^ In addition, how much of the initially generated hot electrons are consumed under a given reaction condition and the relative contributions of different reduction pathways are not well understood either. Earlier pump–probe studies in undoped and Mn-doped QDs under pulsed excitation conditions with ⟨*N*⟩ ≫ 1, where ⟨*N*⟩ is the number of photons absorbed/QD by each pump pulse, measured the yield of e_aq_ surviving initial loss via geminate recombination by monitoring their absorption.^11,12^ In these studies, the absorbed photon-to-e_aq_ yield at nanoseconds after photoexcitation was determined to be several percent, depending on the excitation fluence of the pump pulse. Although only a part of the initially produced hot electrons is ultimately detected as e_aq_, these studies provide useful insights into the hot electron production efficiency under pulsed excitation. Under weak cw excitation, where the competitive kinetics of the processes involving hot electron upconversion are different from those under pulsed excitation conditions (e.g., multiexciton decay), optical detection of e_aq_ is even more difficult due to its low time-averaged absorption intensity. Furthermore, spectroscopic signatures of the initially produced hot electrons in the QDs and e_pre_ in liquid media are less well defined than those in e_aq_, making it very difficult to quantify the efficiency of hot electron upconversion via optical measurements.

In this study, we determined the approximate quantum yield of generating upconverted hot electrons in Mn-doped CdSSe/ZnS QDs under continuous white-field visible light excitation using a reduction reaction selectively driven by upconverted hot electrons. For this purpose, we employed the reductive dechlorination of monochloroacetate (ClCH_2_CO_2_^–^, MCA) as a model reaction:

$${\mathrm{ClCH}}_{2}{\mathrm{COO}}^{-}+e^{-}\to \cdot {\mathrm{CH}}_{2}{\mathrm{COO}}^{-}+{\mathrm{Cl}}^{-}$$ 1

The reduction of MCA initially produces a carboxymethyl radical anion (·CH_2_COO^–^) and a Cl^–^ ion, as shown in eq 1. The one-electron reduction potential of MCA has been reported to be −2.7 V vs NHE in water.^36^ Therefore, MCA can be reduced by the initially upconverted hot electrons (∼−4 V vs NHE), e_aq_ (−2.9 V vs NHE),^37^ and e_pre_ (more negative than e) but not by the band-edge electrons.^38–41^ By measuring the photon-to-product quantum yield (QY_prod_), under the reaction condition that maximizes the consumption of the initially upconverted hot electrons as well as e_pre_ and e_aq_ derived from hot electrons, we determined the lower limit of the quantum yield for generating the upconverted hot electrons (QY_hot e_) in aqueous media. Under 455 nm excitation, QY_hot e_ for Mn-doped CdSSe/ZnS core/shell QDs synthesized in this work was determined to be as high as 35–40% in an aqueous medium using methanol (10% v/v) as a hole scavenger at an excitation intensity of ∼0.1 W/cm^2^. Considering the relatively low light intensity used to generate the upconverted hot electrons, Mn-doped QDs are a highly efficient source of hot and solvated electrons capable of driving challenging reduction reactions. Our study also shows photoexcitation history-dependent QY_prod_, presumably due to an excitation-induced change of the QD surface charge that affects the local reactant concentration on and near the QD surface, revealing the complex interplay of QD photophysics and reaction kinetics. The findings of this study and the method for quantifying QY_prod_ and QY_hot e_ will be important for the further development of more efficient hot-electron-generating QDs and for optimizing the reaction conditions for hot electron-enabled photocatalysis driven by visible light.

## Results and Discussion

To quantify the quantum yield of generating upconverted hot electrons that can perform reduction in aqueous media, we carried out the reduction of MCA at a pH of 12 using Mn-doped CdSSe/ZnS QDs passivated with mercaptoethanesulfonate (MES) under continuous-wave 455 nm excitation. All QDs used in this study were synthesized using the published procedure in our earlier studies, summarized in the Experimental Section with more details in the Supporting Information.^18,19^ The optical absorption and photoluminescence (PL) spectra of the Mn-doped CdSSe/ZnS QDs used in this study are shown in Figure 1a. In the PL spectra, exciton PL centered around 490 nm in undoped QDs is almost invisible and dominated by the PL from Mn centered at 635 nm due to the efficient sensitization of the Mn dopant by the exciton of the host QDs. The absorption cross section of Mn-doped CdSSe/ZnS QDs at the excitation wavelength (σ_455_) is 1.0 × 10^–15^ cm^2^.^17^ A representative TEM image of Mn-doped CdSSe/ZnS QDs is shown in Figure 1b.

**Figure 1:**
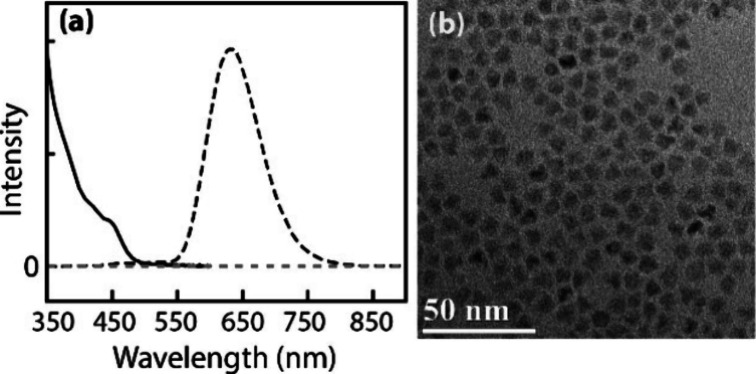
(a) Absorption (solid) and photoluminescence (dashed) spectra of Mn-doped CdSSe/ZnS QDs. (b) TEM image of Mn-doped CdSSe/ZnS QDs.

Figure 2a shows the simplified scheme of the Mn-mediated sequential two-photon hot electron upconversion process in Mn-doped QDs, involving the initial sensitization of Mn^2+^ by an exciton followed by Auger back-energy transfer from the excited state of Mn^2+^ to a second exciton in the conduction band.^29,30^ It also shows the ordering of the energy levels of the conduction band of water, where the hot electrons are initially injected, the standard reduction potential of e_aq_ and the one-electron reduction potential of MCA.^36,38,39^ In the kinetic scheme, *k*_ex_, *k*_1_, *k*_2_, and *k*_Mn_ are the rate constants for the relaxation of the exciton (|ex⟩), sensitization of the Mn excited state (|Mn*⟩) by |ex⟩, Auger back-energy transfer from |Mn*⟩ to an electron in the conduction band to promote it to a hot electron state (|e_hot_⟩), and the relaxation of |Mn*⟩, respectively. Figure 2b illustrates the three possible pathways of MCA reduction by hot electrons from the Mn-doped QDs. The initially upconverted hot electrons, i.e., the electron in the |e_hot_⟩ state before leaving the QD, can reduce the surface-bound MCA via interfacial hot electron transfer. Once the electron in the |e_hot_⟩ state is injected into the conduction band of water, e_pre_ and e_aq_ can reduce MCA via static quenching and diffusive bimolecular reaction, respectively. The hole scavenger (HS) role is to fill holes left behind following the loss of electrons due to reduction reactions.

**Figure 2:**
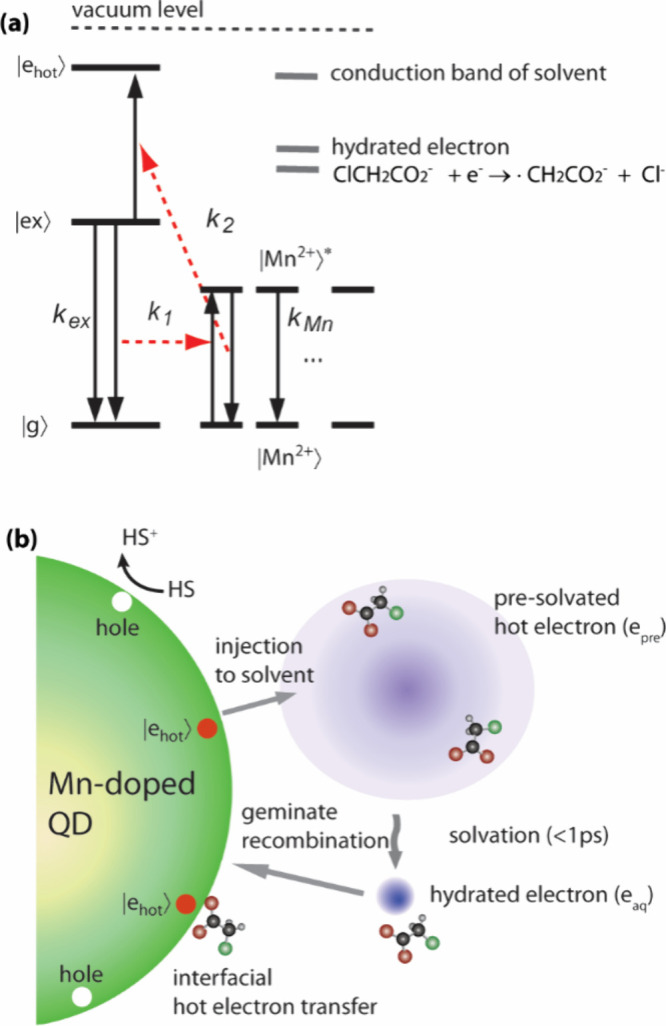
(a) Scheme of Mn-mediated hot electron upconversion. |g⟩, |ex⟩, and |e_hot_⟩ represent the ground state, exciton state, and hot electron state of the host QD, respectively. |Mn⟩ and |Mn*⟩ are the ground and excited states of the Mn dopant, respectively. (b) Illustration of the possible reduction pathways of monochloroacetate (MCA) by hot electrons in QD, presolvated electrons (e_pre_), and hydrated electrons (e_aq_). HS is a hole scavenger.

In an earlier pump–probe study that investigated the quenching of several different forms of e_pre_ produced from UV photoionization of water, it was reported that e_pre_ in the conduction band of water can undergo static quenching.^34^ This process depleted nearly 20% of the population by 0.1 M efficient electron acceptors such as NO_3_^–^ and Cd^2+^ ions. This indicates that e_pre_ derived from the upconverted hot electrons can also be a significant player in photocatalytic reduction reactions using Mn-doped QDs as the hot electron source. In earlier studies, the initial spatial distribution of e_eq_ produced from UV photoionization was empirically modeled to have an exponential form with a width on the order of several nanometers from its origin depending on the energy of the photoionized electrons, which should reflect the spatial extent of e_pre_.^42,43^ If the upconverted hot electrons create an initial spatial distribution on a similar length scale from the surface of the QDs, then the kinetics of the reaction involving |e_hot_⟩ and e_pre_ should be particularly sensitive to the local reactant concentration close to the QDs that may differ from the bulk reactant concentration. Therefore, each pathway illustrated in Figure 2b should depend on the surface adsorption/desorption equilibrium of MCA and the QD-passivating ligand, the number of MCA within the volume of the encounter complex between e_pre_ and MCA, and the bimolecular collision between e_aq_ and MCA.

These reduction pathways involving |e_hot_⟩, e_pre_, and e_aq_ compete with both cooling of |e_hot_⟩ within the QDs and geminate recombination of e_aq_ with the QD. Earlier studies on e_aq_ generated from UV radiolysis of water reported geminate recombination times as fast as 5 ps, which contributes to the loss of initially generated e_aq_.^44^ The extent and time scale of geminate recombination between the QD and e_aq_ are not well known, although its presence has been discussed in earlier studies.^11,12^ However, we consider that it can be minimized more effectively than the case of e_aq_ generated by the photoionization of molecular precursors by combining a sufficiently high concentration of MCA, high pH suppressing the competing reduction of protons, and effective hole scavenging from the QDs. Under the reaction condition that depletes |e_hot_⟩, e_pre_, and e_eq_ primarily through the reduction of MCA by effectively suppressing the competing channels, the extent of MCA reduction provides an estimate of the quantum yield of generating the upconverted hot electrons, as detailed below.

Here, we determined the two key quantities for the reduction of MCA performed using Mn-doped CdSSe/ZnS QDs as the hot electron source at a given excitation intensity: (i) absorbed photon-to-product quantum yield (QY_prod_) under varying reactant concentrations and hole-scavenging conditions and (ii) absorbed photon-to-hot electron quantum yield (QY_hot e_) quantifying the maximum possible number of photogenerated hot electrons usable for the reduction reaction in the solution phase. Since generating one hot electron requires two excitons in Mn-mediated hot electron upconversion, we define QY_prod_ reported in this work as (number of products produced)/(number of photons absorbed/2) such that the value of QY_prod_ is in the 0–1 range (see the Supporting Information). Experimentally, we determined QY_hot e_ by taking the maximum value of QY_prod_ under optimized reaction conditions to minimize the loss of |e_hot_⟩, e_pre_, and e_eq_ via other paths not involving the reduction of MCA. Therefore, QY_hot e_ determined in this way can be interpreted as the lower limit of QY_hot e_ under a given excitation condition, whereas QY_prod_ represents the reductive capability under a given reaction condition.

Throughout this study, QY_prod_ was determined by measuring the concentration of Cl^–^ ions, [Cl^–^], produced via the reductive dechlorination reaction of MCA shown in eq 1 using an ion-selective electrode. ·CH_2_COO^–^ produced along with Cl^–^ is known to undergo subsequent reaction to produce acetate via H abstraction and succinate via dimerization as the products.^40^ For the quantification of the extent of the reaction, Cl^–^ is a more reliable product species since the change in [Cl^–^] has been shown to closely match the change in the concentration of MCA, [MCA], in both an earlier study and our present work.^40^ We verified that the changes in [Cl^–^] and [MCA] agreed within ∼5% by employing multiple quantification methods, including an ion-selective electrode (for [Cl^–^]), mass spectrometry (for [MCA]), and nuclear magnetic resonance spectroscopy (for [MCA] and [Cl^–^]) (see the Supporting Information). The close agreement among these methods validates our choice of Cl^–^ quantification as a reliable and cost-effective approach, enabling the determination of both the reaction kinetics and QY_prod_.

Since the rate of MCA reduction by |e_hot_⟩, e_pre_, and e_aq_ depends on both the MCA concentration and the hole-scavenging conditions, we quantified QY_prodd_ at varying initial MCA concentrations ([MCA]_0_) and using several different hole scavengers. Figure 3a compares the time-dependent concentration of Cl^–^ ions produced, [Cl^–^]­(*t*), at four different [MCA]_0_ values using methanol (10% v/v) as the hole scavenger at pH = 12 and an excitation intensity of *I*_455_ = 0.16 W/cm^2^. The concentration of QDs in the reaction mixture was 0.76 μM, giving rise to an absorbance of 0.2 at an excitation wavelength for a path length of 1 cm. This QD concentration was used throughout all of the reactions performed in this study. We chose methanol among different hole scavengers used for this study to examine the dependence on [MCA]_0_ since the quantification of [Cl^–^]­(*t*) with an ion-selective electrode and calibration was the most reliable and faster compared to using other hole scavengers, which are tertiary amines. When undoped CdSSe/ZnS QDs were used instead of Mn-doped QDs, no Cl^–^ was produced as indicated by the black squares on Figure 3a. The [Cl^–^]­(*t*) values measured at *t* = 5 and 120 min from the reaction using undoped QDs at [MCA]_0_ = 0.1 M are shown in Figure 3a for comparison. From the [Cl^–^]­(*t*) values and the number of photons absorbed by the QDs between the two adjacent time points in Figure 3a, QY_prod_(*t*) was calculated and is compared for different [MCA]_0_ values in Figure 3b.

**Figure 3:**
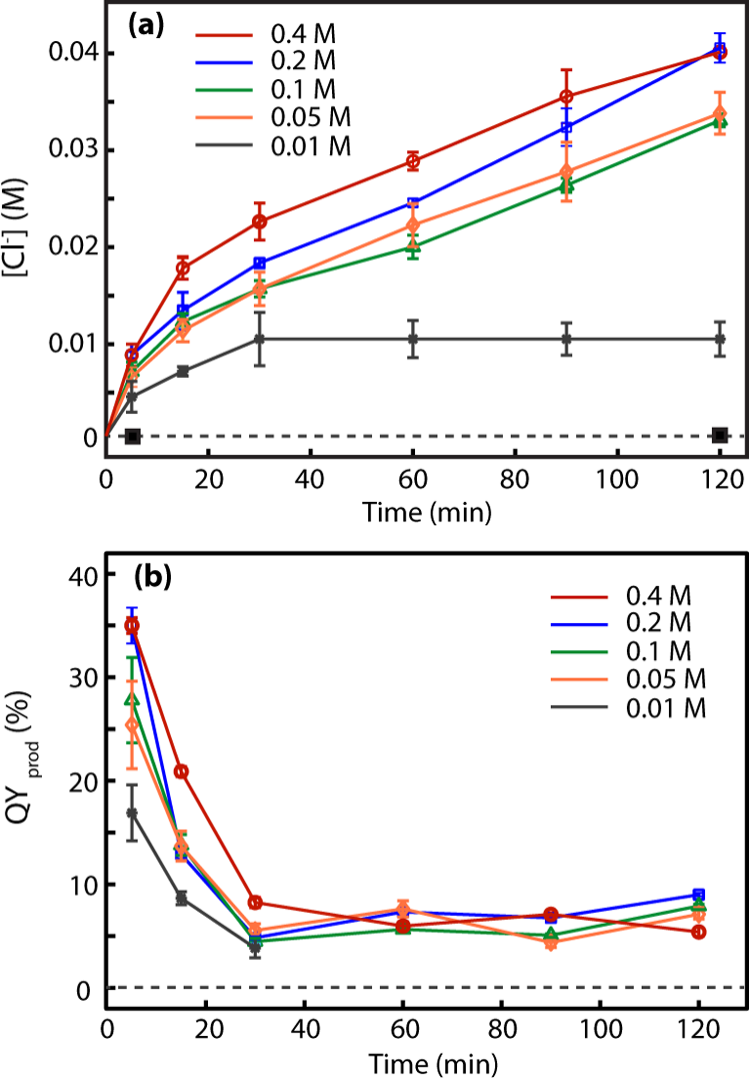
Comparison of (a) [Cl^–^]­(*t*) and (b) QY_prod_(*t*) from the reactions with different [MCA]_0_. In panel (a), [Cl^–^]­(*t*), the reaction using undoped CdSSe/ZnS QDs is shown for [MCA]_0_ = 0.1 M at *t* = 5 and 120 min indicated with a marker (black square). In panel (b), QY_prod_(*t*) for [MCA]_0_ = 0.01 M is shown only up to 30 min since the reaction completes in 30 min. All reactions were performed using methanol (10% v/v) as the hole scavenger at a pH of 12 and at an excitation intensity of *I*_455_ = 0.16 W/cm^2^.

In Figure 3a, [Cl^–^]­(*t*) increases with increasing [MCA]_0_ as anticipated, while it exhibits a saturation at higher [MCA]_0_. Interestingly, QY_prod_ (*t* = 5 min) calculated for the time interval between 0 and 5 min is substantially higher than those at later time intervals. QY_prod_ (*t* = 15 min) for the time interval between 5 and 15 min decreased to about half of QY_prod_ (*t* = 5 min). At later times, QY_prod_(*t*) appears to reach a steadier value. This abrupt decrease in QY_prod_ during the early times of the reaction cannot be explained by the decrease in [MCA]­(*t*) during the reaction, which is relatively a small change, e.g., < 6% change of [MCA]­(*t*) between *t* = 0 and 30 min for [MCA]_0_ = 0.4 M. In principle, one can interpret the change in the values between QY_prod_ (*t* = 5 min) and QY_prod_ (*t* > 5 min) as the result of the QD’s decreased capability of producing hot electrons or/and the reduced local concentration of MCA at the site of reaction, i.e., on the surface and in the vicinity of the QDs.

While the exact reason is unclear, we conjecture that excitation-induced change in the surface charge affecting the local concentration of MCA on and near the QDs may be mainly responsible for the decrease in QY_prod_ on the time scale of tens of minutes based on several recent studies. Gamelin and co-workers reported that electron trapping upon photoexcitation occurrs on the time scale spanning milliseconds to hours, revealing the presence of slow pathways accumulating the electrons at trapping sites in CdSe QDs.^45^ Cossairt and co-workers also reported that the accumulation of electron charge can occur on the time scale of minutes upon photoexcitation in CdS QDs, which dissipates on a similar time scale when the photoexcitation stops.^46^ We consider that such slow charging of the QD surface is also plausible in the photoexcited Mn-doped QDs since part of the photogenerated electrons can still thermalize to the band edge. Under such conditions, it would create a less favorable electrostatic interaction between the QD and the negatively charged MCA at the length scale of the Debye length from the surface of the QDs compared to before excitation. In aqueous media at the ionic strength comparable to the present experimental condition of pH = 12, the Debye length is several nanometers that can affect the interfacial hot electron transfer and reduction by e_pre_.^47^ Therefore, the possible decrease in the local concentration of MCA would particularly affect the reduction pathways involving |e_hot_⟩ and e_pre_, whereas reduction by e_aq_ diffusing into the bulk solution may be less affected.

To test whether the hypothesized excitation-induced change in the local concentration of MCA that may have resulted in the change of QY_prod_ can be reverted, we compared two values of QY_prod_(*t*) with 5 min of photoexcitation, QY_prod_ (*t* = 5 min), to the same solution separated by a 6 h light-off period, QY_prod_ (*t* = 5 min + 6 h off + 5 min). The light-off period inserted between the two measurements was intended to revert any photoexcitation-induced changes to the state before photoexcitation. The comparison was made at [MCA]_0_ = 0.1 M and *I*_455_ = 0.16 W/cm^2^ using methanol (10% v/v) as the hole scavenger. The ratio of two QY_prod_(*t*) values is $\frac{{\mathrm{QY}}_{\mathrm{prod}}(t=5\;\min s+6\;h\;\mathrm{off}+5\;\min)}{{\mathrm{QY}}_{\mathrm{prod}}(t\;=\;5\;\min)}=1.05$ , showing only a 5% relative difference between the two excitation conditions. In contrast, for the continuous excitation without a 6 h off period, the ratio of two QY values is $\frac{{\mathrm{QY}}_{\mathrm{prod}}(t\;=10\;\min)}{{\mathrm{QY}}_{\mathrm{prod}}(t=5\;\min)}=0.49$ . This result clearly shows the presence of the excitation history dependence of QY_prod_, which can be reversed by introducing a “dark” period. We also compared the product yields from the reactions with 30 min of continuous photoexcitation and six cycles of 5 min on/5 min off sequence as shown in the following expression. The ratio of two QY values is $\frac{{\mathrm{QY}}_{\mathrm{prod},\mathrm{on}/\mathrm{off}}}{{\mathrm{QY}}_{\mathrm{prod},\mathrm{continous}}}=1.1$ . The latter excitation scheme resulted in only a moderate increase (∼10%) of the product yield compared to continuous excitation. This indicates that a substantially longer time than 5 min is required for the reversal of the excitation history dependence.

In addition to the possible excitation-induced charging, Kobayashi and co-workers reported that the excitation-induced change in the ligand adsorption/desorption equilibrium on the QD surface can also alter the reduction reaction.^14^ In their study of the reduction of perfluorooctanesulfonate (PFOS) using hot electrons produced by the Auger process in CdS QDs, photoinduced desorption of the ligand (mercaptopronionic acid, MPA) passivating the QDs facilitates the adsorption of PFOS. They observed that inserting the “light-off” periods of 10 s to a minute in duration during the photoexcitation led to reduced product yield by allowing the readsorption of MPA, which inhibited the binding of PFOS on the QD surface. Interestingly, their observation of the excitation history dependence is opposite to ours, and the time scales are much shorter (tens of seconds vs tens of minutes). These differences may arise from different dielectric properties and QD surface-binding affinities of the different reactants. In our case, the more polar, weakly binding MCA may be more affected by surface charging than by photoinduced ligand desorption, whereas PFOS may be more sensitive to the interfacial ligand adsorption/desorption equilibrium. A detailed understanding of the kinetics and efficiency of the hot electron-enabled reduction using the upconverted hot electrons from Mn-doped QDs may be reactant-specific, depending on, e.g., the charge of the reactant and QD surface-binding affinity, and will require further studies. Nevertheless, the results illustrate the complex interplay among the factors determining the reaction kinetics, including the photoexcitation, surface charging, binding, and access of the ligand and reactant, and the role of hole scavengers that will be discussed shortly below.

Regardless of the origin of the higher QY_prod_ (*t* = 5 min) than the values at later times, the maximum value of QY_prod_ that saturates at the highest [MCA]_0_ corresponds to the reaction condition, where all the photogenerated |e_hot_⟩, e_pre_, and e_aq_ are maximally depleted by MCA. Therefore, we can take the maximum saturated value of QY_prod_ as QY_hot e_ for the specified excitation intensity and hole scavenger. For the Mn-doped QDs used for the measurements shown in Figure 3, QY_hot e_ determined at *I*_455_ = 0.16 W/cm^2^ is 35%, which corresponds to the value of QY_prod_ (*t =* 5 min) at [MCA]_0_ = 0.4 M. Considering the use of relatively low-intensity cw visible light for hot electron upconversion, QY_hot e_ is remarkably high and exceeds the performance of other hot electron/solvated electron sources operating under visible and even some sources under UV excitation. For example, earlier studies using the UV photoionization of sulfite ions reported the quantum yield of producing e_aq_ to be 11.6% with 254 nm excitation, while it increases to 23% with 200 nm excitation.^40,48^ Information on the quantum yield of producing hot electrons is scarcer for the systems driven by visible light, which are generally less efficient than UV-photoionized systems. In one study, an iridium complex, *fac*-tris­(2-phenylpyridine)­iridium (Irppy), which produces e_aq_ with visible light (532 nm) from its triplet state (^3^Irppy) prepared by another photoexcitation at 430 nm, reported a quantum yield of 1.3%.^49^

Since maintaining redox balance is crucial to sustain the production of hot electrons via upconversion through the process depicted in Figure 2, efficient hole scavenging is necessary for sustained hot electron-enabled reduction. In Figure 4a,b, we compare the dependence of [Cl^–^]­(*t*) and QY_prod_(*t*) on the hole-scavenging condition for the reactions with [MCA]_0_ = 0.1 M and *I*_455_ = 0.16 W/cm^2^ at pH = 12. Here, we compared tris­(2-aminoethyl)­amine (TAEA, 0.1 M), triethanolamine (TEOA, 0.1 M), and methanol (10% v/v) as hole scavengers, which have been frequently used in photocatalysis using QDs of varying structures, including core only, core/shell, doped, and undoped QDs.^14,18,21^ Among the amine-based hole scavengers tested, TAEA showed significantly higher QY_prod_ than TEOA at the same concentration of 0.1 M, although both were reported to be effective hole scavengers for CdS QDs used for the photocatalytic reduction reactions.^5,14,21^ Interestingly, TEOA provided significantly better colloidal stability of the Mn-doped CdSSe/ZnS QD than TAEA and methanol, keeping the QDs well suspended throughout the entire reaction, whereas a slight turbidity of the solution developed over the course of the reaction for TAEA and methanol. This suggests that stronger binding of TEOA on the ZnS shell surface may impede the access of MCA to the surface of the QDs, thereby diminishing QY_prod_ despite their efficient hole-scavenging capability. Triethylamine (TEA) is another hole scavenger used in the earlier studies of the upconverted hot electron-enhanced photocatalytic reduction reactions. However, a much higher concentration (∼1 M) was required to have QY_prod_ values comparable to those obtained with TAEA and TEOA, exhibiting weaker hole-scavenging capability than other amine-based hole scavengers studied here (Supporting Information).

**Figure 4:**
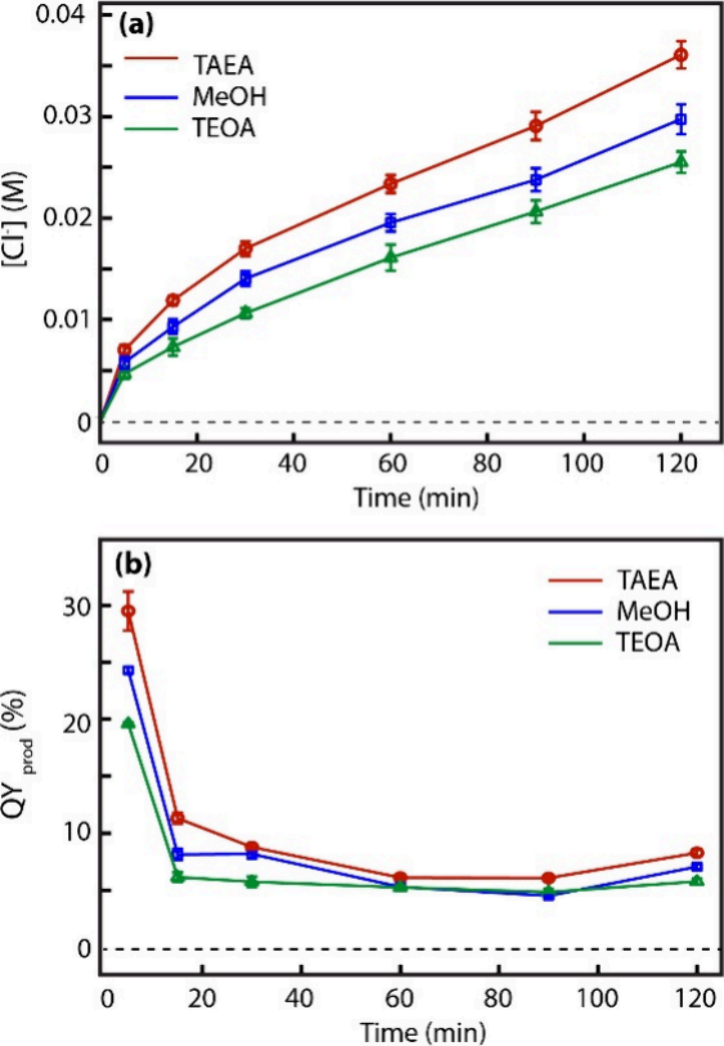
Comparison of (a) [Cl^–^]­(*t*) and (b) QY_prod_(*t*) from reactions with different hole scavengers. All reactions were performed with [MCA]_0_ = 0.1 M, pH 12, and at an excitation intensity of *I*_455_ = 0.16 W/cm^2^.

Since the Mn-mediated hot electron upconversion described in Figure 2a is a sequential biphotonic process, the generation of hot electrons is expected to follow a quadratic excitation intensity dependence.^17,23^ In our earlier studies of hot electron photoemission in vacuum and the hot electron-enhanced H_2_ production in water using Mn-doped QDs, we observed a quadratic excitation intensity dependence at low intensities for both studies, consistent with biphotonic hot electron upconversion.^17,23^ At the higher excitation intensities, switching to the linear excitation intensity dependence was observed for both, indicating the saturation of the population of one of the species (electron or Mn excited state) involved in the upconversion process, while the switching occurred at different intensity regimes. H_2_ production by Mn-doped QDs dispersed in aqueous solution showed the transition to linear excitation intensity dependence at an intensity of ∼0.4 W/cm^2^ (Xe light), whereas hot electron photoemission in vacuum maintained a quadratic intensity dependence to tens of W/cm^2^ (405 nm cw laser light). In contrast to these observations, Cao and co-workers recently reported that the hot electron-enabled organic reactions in polar organic solvents performed using Mn-doped CdS/ZnS QDs showed the linear excitation intensity dependence of the reaction rate within the studied intensity range (0.02–0.3 W/cm^2^) with no indication of biphotonic behavior.^22^ These observations suggest that the photon order of upconverted hot electron-enabled reduction reactions is sensitive to the local environment of the QDs, reaction phase (solution vs interface), solvent, and the population dynamics of intermediate states involved in the upconversion process.

To investigate the photon order of hot electron generation under the reaction conditions of this study, we measured QY_prod_ as a function of the excitation intensity. Figure 5a shows the excitation intensity dependence of [Cl^–^]­(*t* = 5 min) for reactions performed with [MCA]_0_ = 0.1 M and methanol (10% v/v) as the hole scavenger in the intensity range of 0.03–1.0 W/cm^2^. The corresponding QY_prod_ (*t* = 5 min) values are plotted in Figure 5b. QY_prod_ (*t* = 5 min) increases with excitation intensity in the 0.03–0.10 W/cm^2^ range indicating the photon order larger than 1, but its increase is sublinear, i.e., the photon order is between 1 and 2. At higher intensities, QY_prod_ (*t* = 5 min) decreases with increasing excitation intensity. Clearly, the kinetics of reduction of MCA by upconverted hot electrons from Mn-doped QDs in the solution phase are more complex than the kinetic model for hot electron upconversion shown in Figure 2a can capture. That model better explains the quadratic intensity dependence of photoemitted electrons in vacuum, where the system is free from complications such as diffusion of reactants/products and interfacial adsorption/desorption dynamics at the QD surface.^15,23^ The significant deviation from quadratic behavior, along with the excitation history-dependent QY_prod_ of MCA reduction, suggests that photogeneration and consumption of hot electrons in the solution phase are governed by multiple factors in a complex manner. Not only the process of hot electron upconversion within the QD, but the interplay between QD surface photochemistry and diffusive reaction kinetics in solution must also be considered. All these factors influence the overall effectiveness of upconverted hot electrons for the reduction reaction and may necessitate reaction-specific strategies to maximize the reduction efficiency for a given QD structure and reaction condition.

**Figure 5:**
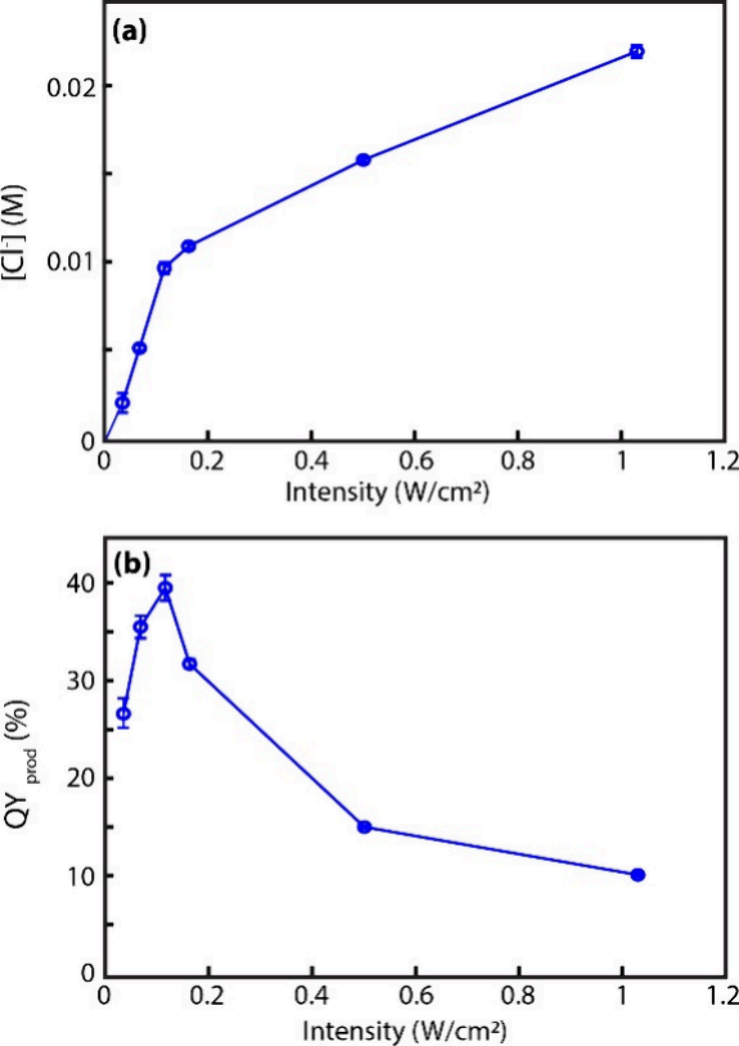
Excitation intensity dependence of (a) [Cl^–^]­(*t =* 5 min) and (b) QY_prod_ (*t =* 5 min). All reactions were performed with [MCA]_0_ = 0.1 M and methanol (10% v/v) as the hole scavenger at pH = 12.

## Conclusions

In this work, we quantified the quantum yield of generating upconverted hot electrons from Mn-doped CdSSe/ZnS QDs via the Mn-mediated Auger process under weak cw visible light excitation (455 nm). Although recent studies have demonstrated the remarkable capability of upconverted hot electrons from Mn-doped QDs to drive challenging reduction reactions, their generation efficiency has remained largely uncharacterized. We measured the absorbed photon-to-product quantum yield (QY_prod_) for the reductive dechlorination of MCA chosen as a hot electron-selective reaction to quantify the reductive capability of the upconverted hot electrons under various excitation intensity and hole-scavenging conditions. From the saturation value of QY_prod_, obtained under conditions that maximize depletion of hot electrons, presolvated electrons, and hydrated electrons via MCA reduction, we determined a lower limit for the absorbed photon-to-hot electron quantum yield (QY_hot e_). In Mn-doped CdSSe/ZnS QDs studied here, QY_hot e_ reached 35–40% at an excitation intensity of ∼0.1 W/cm^2^, exceeding the performance of many other hot electron generation methods. Interestingly, QY_prod_ exhibited a dependence on the excitation history, suggesting a possible role of the surface charge-modulated local reactant concentration. Moreover, a nonmonotonic dependence of QY_prod_ on excitation intensity deviating from the expected quadratic scaling for biphotonic upconversion was observed. These observations highlight the complexity in the effectiveness of the upconverted hot electrons for the reduction reaction in solution, which is governed not only by the photophysics of hot electron upconversion within the QD but also by the interfacial and diffusive chemical process on the QD surface and in the solution phase. The insights gained in this study offer valuable guidance for designing more efficient photocatalytic systems based on hot electrons upconverted in colloidal QDs.

## Experimental Section

### Synthesis of the QD Photocatalyst

Mn-doped and undoped CdSSe/ZnS core/shell quantum dots (QDs) were synthesized using a previously reported procedure.^18,50^ The detailed synthesis procedures are provided in the Supporting Information. Briefly, the CdSSe core QDs (3.6 nm in diameter) were synthesized to target an exciton absorption peak at 450 nm, which is sufficiently high in energy to ensure initial sensitization and is readily accessible using a light-emitting diode. A ZnS shell was coated onto the core with a thickness of 1.8 nm, and Mn^2+^ ions were doped into the shell. The core/shell structure with the ZnS shell provided sufficient protection against degradation of the QDs during the hot-electron-induced reduction reaction.

### Photocatalytic Reduction of Monochloroacetate (MCA)

All photocatalytic reduction reactions of MCA by Mn-doped and undoped CdSSe/ZnS QDs were performed in aqueous solution at the same QD concentration (0.76 μM), corresponding to an absorbance of 0.2 at the excitation wavelength (455 nm) for a 1 cm path length. To adjust the solution pH to 12, a 0.6 M NaOH solution was added as needed. Each reaction was conducted in either a standard 8 mL glass vial or a quartz cuvette, which served as the reactor vessel and was immersed in a water bath connected to a circulating chiller to maintain a constant temperature of 17 °C during photoexcitation. Prior to the reaction, each sample solution was purged with N_2_ to remove dissolved oxygen, which could interfere with the reduction of MCA by hot electrons and solvated electrons. A cannula was attached between the vial (or cuvette), and a 25 mL three-neck flask was maintained under a slight positive pressure of N_2_ to prevent excessive pressure buildup within the reactor vessel during the reaction. A 455 nm light-emitting diode (LED) was used as the light source for all reactions conducted at a fixed light intensity of 0.16 W/cm^2^, which illuminated the entire volume of the reaction mixture. For the intensity-dependent measurements, two 455 nm diode lasers mounted on a translation stage were used to vary the light intensity at the reactor location within the range of 0.03–1.0 W/cm^2^. Light intensity was measured using a Si-photodiode-based optical power meter (Thorlabs). See the Supporting Information for additional details on the reactor setup.

### Quantification of the Reaction Product

Quantifying the concentration of Cl^–^ at time *t*, [Cl^–^]­(*t*), in the reaction product was carried out using a Cl^–^ ion-selective electrode. Aliquots were taken from the reactor vessel at desired time points using a N_2_-purged syringe and stored in capped 5 mL centrifuge tubes for quantification. The concentration of Cl^–^ produced from the reduction reaction was quantified by using a chloride ion-selective electrode (HI 4107, Hanna Instruments). All the aliquots taken were initially centrifuged to precipitate the QDs. A fixed volume of supernatant solution was taken using a micropipette and diluted to the mixture of an ionic strength adjuster (HI-4000-00) and deionized water to a known dilution factor. A calibration curve used for the determination of [Cl^–^]­(*t*) in the reaction product was constructed using standard solutions of known [Cl^–^] prepared in a solvent matrix matching the composition of the solvent used for each reaction (i.e., mixture of water, ionic strength adjuster, and corresponding hole scavenger). For each reaction performed with a different hole scavenger, a separate calibration curve was used for quantification. The [Cl^–^]­(*t*) values and error bars reported in this work are based on three independent reactions for each reaction condition and three independent measurements with the ion-selective electrode for each reaction. Background Cl^–^ signals that may be present in the reaction mixture were subtracted by measuring [Cl^–^] before photoexcitation in all measurements. Additional quantification methods using mass spectrometry and NMR are described in the Supporting Information.

## Supplementary Material



## Notes

Chemicals, synthesis procedures, quantification details, calculation of quantum yields, and reactor design (PDF)
