# Supplementary material for: Determination of the Generation Efficiency and Reductive Reactivity of the Upconverted Hot Electrons from Mn-Doped Quantum Dots under Weak Visible Light
Source: J Am Chem Soc. 2025 Oct 2;147(41):37497–505. doi: 10.1021/jacs.5c12004 (PMC12532206; doi:10.1021/jacs.5c12004)
Supplement: Supplementary file 1 [file ja5c12004_si_001.pdf]

Supplementary Information

Determination of the generation efficiency and reductive reactivity of the upconverted hot electrons from Mn-doped quantum dots under weak visible light

Connor Orrison[1], Ian C. Schulze[1], Zefan Zhang[1], Dallas Freitas[1], Ian Murray[1], Xin Yan[1], Christian Hilty[1] and Dong Hee Son[1,2,3]

[1]Department of Chemistry, Texas A&M University, College Station, Texas 77843, United States
[2]Department of Physics and Astronomy, Texas A&M University, College Station, TX 77843, USA
[3]Center for Nanomedicine, Institute for Basic Science and Graduate Program of Nano Biomedical Engineering, Advanced Science Institute, Yonsei University, Seoul 03722, Republic of Korea

Table of Contents

1. Chemicals --S2
2. Material synthesis ---S2
3. Sample characterization --S3
4. Quantification of the reactant consumption and product production ------S3
5. Calculation of the quantum yield ---S5
6. Reactor --S6
7. Additional data --S7

1. Chemicals

Sulfur (99.998%, Sigma Aldrich), Selenium (99.99% Sigma Aldrich), Cadmium oxide (CdO, 99.99% Sigma Aldrich), Zn stearate (90%, Sigma Aldrich), Manganese acetate (98%, Sigma Aldrich), Oleic Acid(90%, Technical Grade, Sigma Aldrich), 1-Octadecene (90%, Technical Grade, Sigma Aldrich), Oleylamine (70%, Technical Grade, Sigma Aldrich), Tri-n-butyl phosphine (95%, Alfa Aesar).

2. Material synthesis

Synthesis of Mn-doped CdSSe/ZnS QDs

CdSSe Core synthesis: The sulfur (S) precursor was prepared by dissolving 0.08 g sulfur in 10 mL of 1-octadecene. The selenium (Se) precursor consisted of 1.58 g Se powder dissolved in tri-n-butyl phosphine. To prepare the S/Se precursor, 0.475 mL of the Se precursor was injected into the S precursor, which had been heated to 100 °C. The cadmium (Cd) precursor was made by adding 0.128 g of cadmium oxide (CdO) to a mixture of 12 mL 1-octadecene and 2.1 mL oleic acid, then heating it under nitrogen atmosphere to 240 °C. At this point, 2 mL of the S/Se precursor was injected and allowed to react for 4 minutes. The reaction was then rapidly cooled to quench it. The resulting CdSSe core QDs were precipitated using acetone and centrifugation. The core QDs were then redispersed in a small volume of toluene and reprecipitated using methanol. This suspension and precipitation step was repeated a total of three times to ensure removal of excess reactants. The final pellet of core QDs was suspended in toluene.

ZnS shelling and Mn-doping: Two layers of ZnS shell were added to the core QDs using the successive ionic layer adsorption and reaction (SILAR) method. The Zn precursor was a solution of 0.25 M zinc stearate in anhydrous toluene with 5 % 1-octylamine. The core QDs were suspended in 6 mL of 1-octadecene and 2 mL of oleylamine and heated to 220 °C under nitrogen. The S precursor was added dropwise over 3 minutes and allowed to react for 10 minutes. Then, the Zn precursor was added dropwise over 3 minutes and allowed to react for another 10 minutes. This cycle of S precursor followed by Zn precursor was repeated once more. The resulting QDs were cleaned using the same method as for the core QDs, except that a minimal amount of 1-octylamine was included in addition to toluene, which were subsequently used for Mn doping and additional coating with ZnS shell. The cleaned QDs with two ZnS layers were suspended in 6 mL of 1-octadecene and 2 mL of oleylamine and heated to 260 °C under nitrogen. The Mn precursor was prepared by dissolving 28 mg of Mn(II) acetate tetrahydrate in 6 mL of oleylamine under nitrogen to prevent oxidation. This Mn precursor was added dropwise over 3 minutes and allowed to react for 20 minutes. The reaction was then quenched by rapid cooling and washed twice using the same method as for the core QDs. Immediately after Mn doping and cleaning, four additional ZnS layers were added using the SILAR method described above. These QDs were again cleaned using the same procedure as after the first two ZnS layers and resuspended in 5 mL of toluene. To render the

completed QDs water-soluble, a ligand exchange was performed, replacing the native oleylamine ligands with 2-mercaptoethanesulfonate (MES). The oleylamine ligands were stripped by adding 0.3 mL of the completed QDs to 3.5 mL of a 75% chloroform / 25% methanol solution, along with 10 drops of aqueous 6 M NaOH. The aqueous layer was removed and centrifuged. The resulting pellet was sonicated in methanol to redisperse it. The QD/methanol suspension was then transferred to a round-bottom flask containing 10 mL methanol and 1 mL aqueous 6 M NaOH and heated to 50 °C under nitrogen for 8 hours. Afterward, the suspension was centrifuged, the supernatant was decanted, and the resulting pellet was suspended in water.

Synthesis of undoped CdSSe/ZnS QDs

The synthesis procedure of undoped CdSSe/ZnS QDs is identical to that of Mn-doped QDs except the steps of doping Mn is omitted.

3. Sample characterization

Transmission electron microscopy (TEM) images of the QDs dispersed on a TEM grid were obtained on a FEI-Tecnai G2 F20 ST FE-TEM electron microscope. The absorption spectrum of the quantum dots was obtained using a CCD absorption spectrometer (Ocean Optics). The photoluminescence spectra were collected using a fiber-coupled CCD fluorescence spectrometer (QE65pro, Ocean Optics).

4. Quantification of the reactant and product using mass spectrometry (MS) and nuclear magnetic resonance (NMR)

The close matching of the changes in the concentrations of monochloroacetate (MCA) consumed and Cl^- produced was established by comparing the concentrations of MCA ([MCA]) and Cl^- ($[Cl^-]$) before and after the reaction using multiple quantification methods. Description of the methods using MS and NMR are described below.

Quantification of MCA and Cl^- by NMR

For NMR quantification, 20 mM standards were prepared in D_2O and photocatalysis experiments were conducted in D_2O with all other conditions remaining the same as the reaction carried out in H_2O described in the main text. A 0.5 ml aliquot was taken at desired reaction times, centrifuged to remove QDs, and were diluted 3 times prior to quantification. The ^1H NMR experiments, quantifying [MCA], were performed on an Avance 500 spectrometer with a TCI cryoprobe (Bruker Biospin). A pulse sequence with W5 WATERGATE[41] to suppress residual water solvent signal was used with bandwidth between two null points of 1.8 kHz, without decoupling. The pulses were applied with γB_1 of 25.253 kHz at 4.70 ppm, and the spectral width was 20.49

ppm. The recovery delay between scans was 10 s. The ^{35}Cl NMR experiments quantifying [Cl^-] were measured on an AVANCE III 400 MHz spectrometer with a BBO probe (Bruker Biospin). The pulses were applied with γB_1 of 4.7619 kHz at 0 ppm, and the spectral width was 597.8 ppm. The acquisition time was 128 ms and the recovery delay between scans was 10 ms. [MCA] and [Cl^-] values before and after the reaction were determined using the standard solution samples with known concentration. Figure S1(a) shows the comparison of ^1H NMR peaks corresponding to MCA before and after the reaction. Figure S1(b) shows the comparison of ^{35}Cl NMR peaks corresponding to Cl^-. The changes in [MCA] and [Cl^-] match within 5 %.

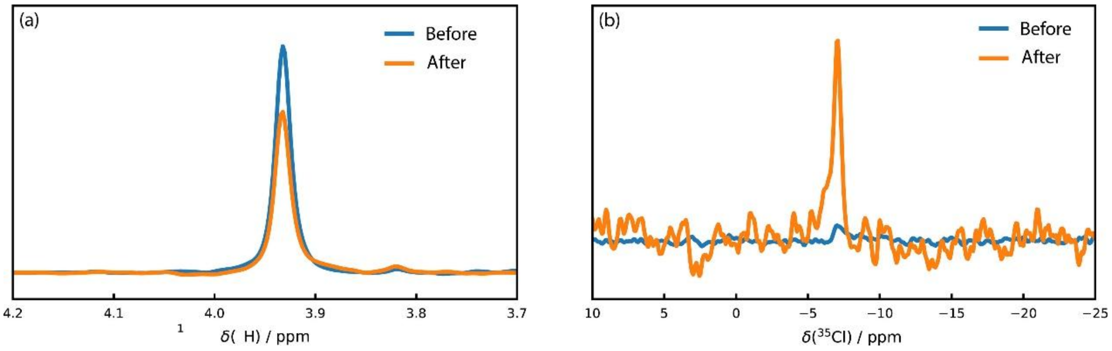

Figure S1. (a) ^1H NMR peaks corresponding to MCA, (b) ^{35}Cl NMR peaks corresponding to Cl^- before and after the reaction. ^{35}Cl NMR spectra before and after the reaction were taken with different number of scans, therefore have different signal/noise ratio.

Quantification of MCA by MS

MS was used to quantify the consumption of MCA during the reduction reaction. Data were acquired using a Thermo Scientific LTQ XL Mass Spectrometer (San Jose, CA) set with the following parameters: in full-scan MS, a mass range of *m/z* 50-250 was used in negative ion mode with a scan rate of 2 microscans at 200 ms. For the atmospheric pressure ionization (API) source, the capillary temperature was set to 275 °C with a capillary voltage of -49V and a tube lens voltage of -68V. For samples analyzed via nano-electrospray ionization (nano-ESI) MS, the samples were placed under Ar and 5 µL aliquots were taken in triplicate at desired intervals and diluted in methanol. Diluted samples were stored in 1 mL capped centrifuge tubes prior to quantification. For sample analysis, bulk solutions were diluted 1000 times and transferred to a 96-well plate (Corning Incorporated; Corning, NY). Ionization of samples was performed using an Advion Biosciences TriVersa NanoMate (Ithaca, NY) for high-throughput chip-based nano-ESI analysis. A spray DC voltage of -1.5 kV was used in negative ion mode analysis, with a backpressure of 0.30 psi. MCA solution was kept under an inert Ar atmosphere with a positive pressure of ~5 psi. Sampling was done dropwise using a 260 µm ID/360 µm OD fused silica capillary (BGB; Alexandria, VA) inserted into the solution. Sampling was done in sets of triplicates. Before analysis, diluted samples

were spiked with standard benzoic acid at a concentration of 50 µM. Average analyte ion intensity was normalized using benzoic acid to minimize sampling variation between wells and nano-ESI emitters. Figure S2 compares the MS spectra before and after the reaction. [MCA] values before and after the reaction was determined using the standard solution samples with known concentration. The change in [MCA] determined by MS agreed with the change in [Cl^-] determined by ion-selective electrode within ~5 %.

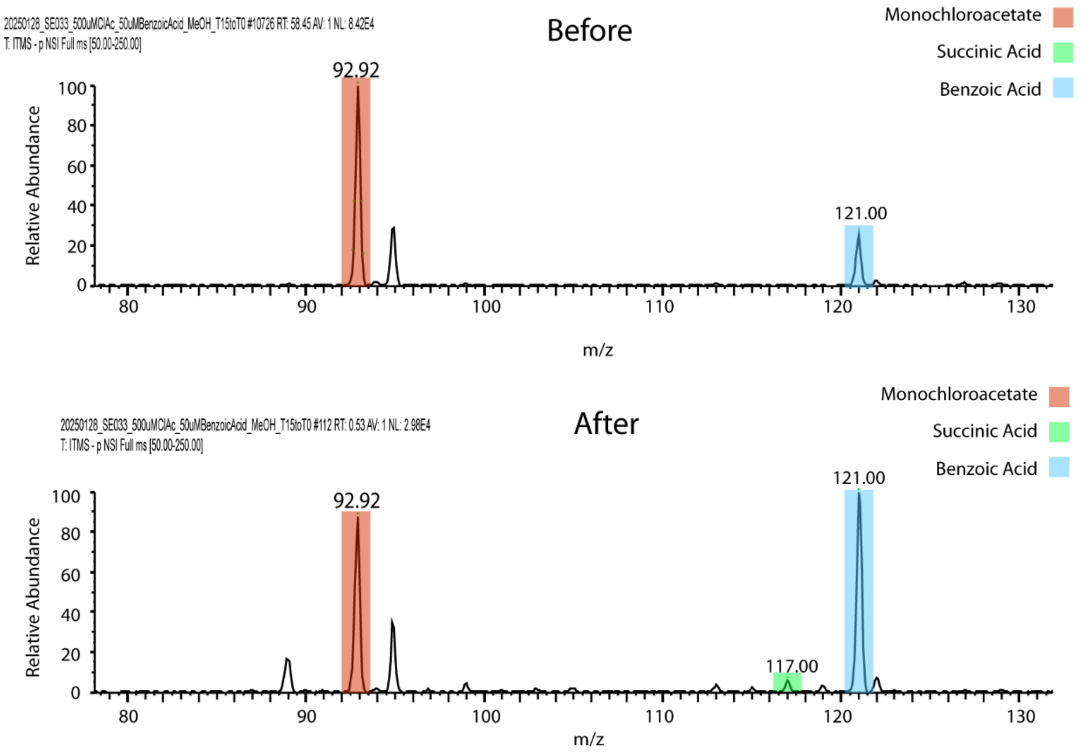

Figure S2. MS spectra of MCA before and after the reaction.

5. Calculation of absorbed photon-to-product quantum yield (QY_{prod})

Photon-to-product quantum yield for a given reaction was calculated as follows, since the production of one hot electron requires two excitons in Mn-mediated hot electron upconversion.

$$QY_{prod} = \frac{\text{\# of Cl}^- \text{ produced}}{\text{\# of photons absorbed}/2}$$

$$\# \text{ of photons absorbed} = \frac{I_{455}\left(\frac{W}{cm^2}\right) \times reacton\ time(s) \times Area(cm^2) \times average\ absorptance}{E_{ph}(J)}$$

$$\# \text{ of } \mathrm{Cl}^- \text{ produced} = [\mathrm{Cl}^-](\mathrm{M}) \times V(L) \times N_A$$

Absorptance is the fraction of incident photons absorbed. For a flat-surfaced reactor vessel (e.g., a cuvette), absorptance is given by $1-10^{-A}$, where A is the absorbance. For a curved reactor vessel (e.g., a vial), the average absorptance was used to account for the variation in sample pathlength along the lateral direction. *Area* is the cross-sectional area of the sample solution in the reactor vessel. E_{ph} is the energy of photon at 455 nm. V is the volume of solution in L, N_A is Avogadro's number.

6. Reactor

The reactor was home-built and consisted of a water bath that maintained a constant temperature for the sample vials using a thermoelectric temperature-controlled circulating water chiller. An air-cooled light-emitting diode (CAHNZON, 455 nm), coupled with an aspheric lens and powered by a constant current source, was used to provide excitation at a fixed intensity of 0.16 W/cm². A single LED could excite two sample vials simultaneously at the same excitation intensity, as confirmed by scanning the intensity profile across the plane of the sample vials using a optical power meter. For higher intensities, two diode lasers, each emitting 4 W output with a defocused beam profile at the sample location, were used to provide up to 1.0 W/cm². The intensity at the sample location was adjusted by changing the distance between the laser and the sample vial.

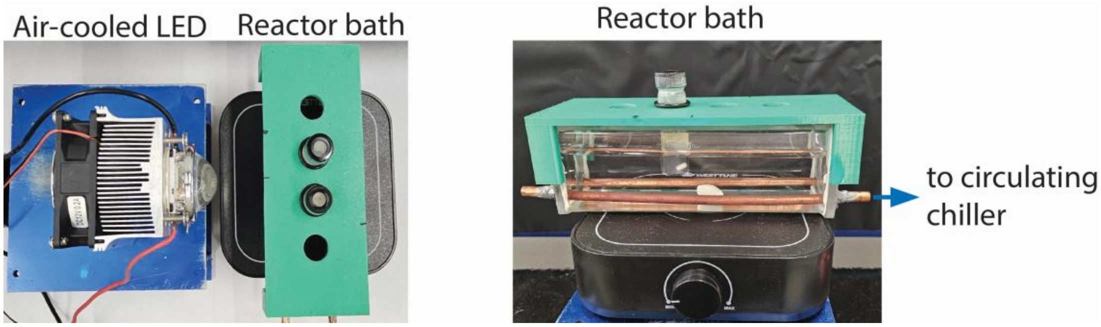

Figure S3. Photographs of the reactor

7. Additional data

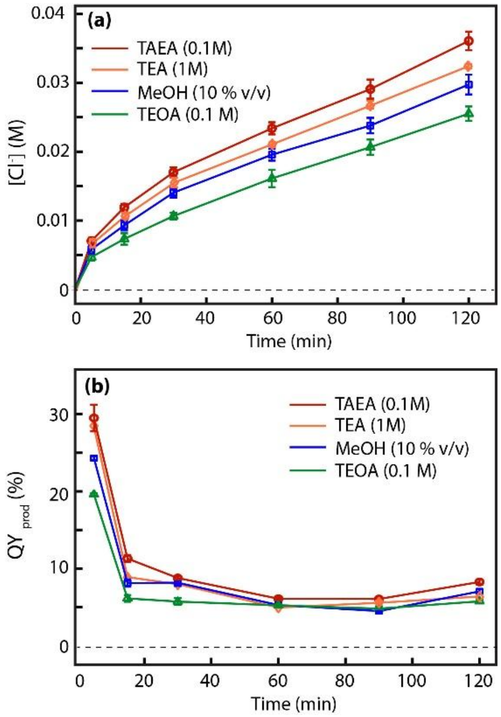

Figure S4. Comparison of (a) $[Cl^-](t)$ and (b) QY_{prod} (t) from the reactions with different hole scavengers. All reactions were performed with $[MCA]_0$=0.1 M, pH of 12 and at the excitation intensity of I_{455} =0.16 W/cm^2. For TEA, much higher concentration was required to exhibit comparable hole scavenging capability to other amine-based hole scavengers.
